# Evolutionary Action Reveals Sex‐Specific Pathogenic and Protective Pathways and Differential Cell Involvement in Alzheimer's Disease

**DOI:** 10.1002/alz70855_101948

**Published:** 2025-12-23

**Authors:** Olivier Lichtarge

**Affiliations:** ^1^ Jan and Dan Duncan Neurological Research Institute, Texas Children's Hospital, Houston, TX, USA; Baylor College of Medicine, Houston, TX, USA

## Abstract

**Background:**

AD incidence is double in female than male, suggesting sex‐specific AD genes remain unknown.

**Method:**

We developed σ_EA_‐Diff, a statistical physics approach exploiting massive evolutionary and phylogenetic sequence data. σ_EA_‐Diff quantifies the selection pressure exerted on genes across populations and identifies those linked to disease risk from significant case‐control differences.

**Result:**

Using 4768 cases and 4689 controls, σ_EA_‐Diff discovered 122 genes (*FDR* 0.05). These candidates interacted mutually (*p* 0.0019), overlapped (*p* 3.10^‐5^) and networked (*z* 7.16) with AD GWAS genes and AD‐related processes (*p* 1.0^‐16^), and were significantly dysregulated in bulk/single cell AD RNAseq (*p* 0.05). In benchmarks comprising the top 122 hits from SKAT‐O and GWAS, σ_EA_‐Diff matched or exceeded performances in network connectivity to known AD genes and processes, and dysregulated genes, revealing novel coding variants carrying risk and functional impact in genes previously associated to AD. Importantly, σ_EA_‐Diff candidates were enriched in modifiers of neurodegeneration in *Drosophila* (*p* 0.05). σ_EA_‐Diff's robust performance in down‐sampling justified sex‐separated analyses, identifying 82 AD candidates in males and 69 in females. Integration with single cell RNAseq revealed cell‐type specific dysregulation of the sex‐specific candidates. Male candidates were significantly dysregulated in microglia and excitatory neurons while female candidates were significant in inhibitory neurons. Integration with neuropathology correlated gene expressions reveals biological processes with sex‐specific enrichment, like the c‐Kit pathway and mRNA splicing. Variant specific analyses revealed certain variants carried differential risk between males and females. APOE4 is known to carry higher risk in females than males, integration of σ_EA_‐Diff genes with APOE genotype revealed candidates potentially underlying this differential risk. Applying the candidates as machine learning features successfully predicted ADs from controls with (AUC 0.83) and without (0.72) APOE genotype information, and especially high accuracy in the tails of the risk distributions.

**Conclusion:**

σ_EA_‐Diff identifies AD genes more robustly than standard methods and predicts new high confidence candidates, including in sex‐specific genes, cell types, pathways and modifiers of neurodegeneration further probing differential effects of APOE. These results point to new potential drug targets and demonstrate the power of quantitative phylogenetics in complex diseases studies.

